# Decoupling Suspension Controller Based on Magnetic Flux Feedback

**DOI:** 10.1155/2013/956790

**Published:** 2013-06-11

**Authors:** Wenqing Zhang, Jie Li, Kun Zhang, Peng Cui

**Affiliations:** College of Mechatronics Engineering and Automation, National University of Defense Technology, Changsha, Hunan 410073, China

## Abstract

The suspension module control system model has been established based on MIMO (multiple input and multiple output) state feedback linearization. We have completed decoupling between double suspension points, and the new decoupling method has been applied to CMS04 magnetic suspension vehicle in national mid-low-speed maglev experiment field of Tangshan city in China. Double suspension system model is very accurate for investigating stability property of maglev control system. When magnetic flux signal is taken back to the suspension control system, the suspension module's antijamming capacity for resisting suspension load variety has been proved. Also, the external force interference has been enhanced. As a result, the robustness and stability properties of double-electromagnet suspension control system have been enhanced.

## 1. Introduction

**Figure 1:**
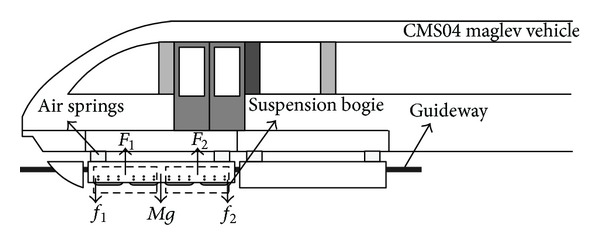
CMS04 mid-low-speed maglev vehicle.

Maglev vehicle has minor noise, no pollution safety, and comfort many other advantages, so the maglev traffic has a bright prospect in the future. Maglev vehicle concludes several complex subsystems [1] with coupling relations. The vehicle's suspension and guidance depend on magnetic force, and its traction relies on linear motion actuator. According to the production method of magnetic force, maglev system can be divided into two types: EDS and EMS. EMS can make vehicle suspend on the guideway keeping a certain gap, but suspension system in itself is not stable, so it needs active control. Maglev control technology is one of crucial techniques in suspension system. Maglev vehicle is a typical nonlinear system, and suspension module concludes two coupled suspension points which are connected by a rigid joint. The coupling problem is not obvious on straight guideway, but when maglev vehicle arrives at the vertical curve and the curved guideway, the coupling problem can make maglev system unstable. So how to work out this problem is an imperative thing for maglev engineers. Lin et al. have studied the design of a robust discrete-time sliding mode control (DT-SMC) [2] for a high-precision electrohydraulic actuator (EHA) recently. Ngoc [3] first proved an explicit criterion for positive linear time-varying differential systems with distributed delay. A new approach can keep stabilization of time-varying nonlinear systems with distributed input delay by feedback of plant's state in paper [4]. The stability bound [5] of the singular perturbation parameter can be obtained by solving a set of matrix inequalities. Control redundancies are proposed [6], for example, the controllable redundancy, observable redundancy, stabilization redundancy, and detectable redundancy. The fuzzy sliding mode control approach [7] can be applied to deal with the output tracking of the uncertain system. Nonlinear value control based on FDM (feedback domination method) is simple and has robustness [8] to some kinds of unknown disturbances. The nonlinear feedback principle is developed [9] using the direct-axis and the quadrature-axis stator voltage as manipulated variables. Roy and Asada have investigated nonlinear feedback control of a gravity assisted under actuated manipulator [10] with application to aircraft assembly. A simple methodology [11] to evaluate harmonic distortion in the frequency domain for circuits and systems made up of a nonlinear high-gain path with a nonlinear feedback network is presented. Think that suspension force is a single function about magnetic flux density, and the relation between suspension force and magnetic density's square is linear [12]. So we take magnetic flux density back to maglev system for promoting the robustness and stability. CMS04 maglev vehicle designed by NUDT (National University of Defense Technology) is showed in Figure 1.

## 2. Foundation of Flux Feedback Control Suspension Model

EMS middle- and low-speed maglev vehicle applies modular suspension bogies, and one module concludes two suspension points. Double suspension points' suspension task is accomplished by one suspension controller. 

Assume the following:

1. the leakage flux of magnetic winding is neglected;
2. the magnetic resistances of the ferrite core and rail are neglected; namely, magnetic potential falls on air gap *z*(*t*) evenly;
3. the inclination angle of magnetic rigid body is minuscule, and active point of magnetic force is considered invariable;
4. the action line of load forces is superposed with direction of suspension gap measured;
5. distribution of mass of the bracket is even, and the masses of two magnets are equal, so the action point of gravity is superposed on the geometry center *O*.

**Figure 2:**
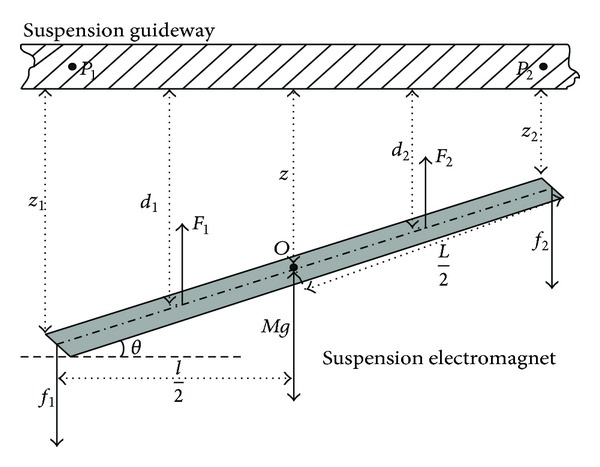
Suspension system's force analysis map.

Based on above assumptions, the force analysis and geometrical relationship of maglev system are showed in Figure 2.

Signs of Figure 2 are illuminated in Table 1.

### 2.1. Mathematical Model

#### 2.1.1. Kinematics Equations

When electromagnet module is in condition of balance, we can obtain the equation ∑*F*_*i*_ = 0 and ∑*M*_*i*_ = 0. The kinematics equations of maglev system can be got as follows:

(1) $$\begin{array}{c} \frac{1}{2}M\cdot \left({\ddot{z}}_{1}+{\ddot{z}}_{2}\right)=Mg-F_{1}-F_{2}+f_{1}+f_{2},\\ \frac{I}{L}\cdot \left({\ddot{z}}_{1}-{\ddot{z}}_{2}\right)=f_{1}\cdot \frac{1}{2}l-f_{2}\cdot \frac{1}{2}l-\frac{1}{4}F_{1}\cdot l+\frac{1}{4}F_{2}\cdot l. \end{array}$$

Extract acceleration signals from (1):

(2) z¨1=−2L−3l2ML·F1+−2L+3l2ML·F2+L+3lML·f1 +L−3lML·f2+g,z¨2=−2L+3l2ML·F1+−2L−3l2ML·F2+L−3lML·f1 +L+3lML·f2+g.

Set *A* = (−2*L* − 3*l*)/2*ML*, *B* = (−2*L* + 3*l*)/2*ML*, *C* = (*L* + 3*l*)/*ML*, and *D* = (*L* − 3*l*)/*ML*.

Simplify (2):

(3) $$\begin{array}{c} {\ddot{z}}_{1}=A\cdot F_{1}+B\cdot F_{2}+C\cdot f_{1}+D\cdot f_{2}+g,\\ {\ddot{z}}_{2}=B\cdot F_{1}+A\cdot F_{2}+D\cdot f_{1}+C\cdot f_{2}+g. \end{array}$$

#### 2.1.2. Electromagnetics Equations

We can get geometrical relationship of parameters in Figure 2. The relation between measurement positions *z*_1_, *z*_2_ and real physical positions *d*_1_, *d*_2_ is

(4) $$\begin{array}{c} d_{1}=\frac{1}{2}\left(z_{1}+z\right)=\frac{3}{4}z_{1}+\frac{1}{4}z_{2},\\ d_{2}=\frac{1}{2}\left(z_{2}+z\right)=\frac{1}{4}z_{1}+\frac{3}{4}z_{2}. \end{array}$$

Magnetic force equations are as follows:

(5) $$\begin{array}{c} F_{1}=\frac{B_{1}^{2}S}{\mu_{0}},\\ F_{2}=\frac{B_{2}^{2}S}{\mu_{0}}. \end{array}$$

#### 2.1.3. Electricity Equations

Voltage balance equations of electromagnet winding are denoted in (6):

(6) $$\begin{array}{c} u_{1}=\frac{2Rd_{1}}{\mu_{0}N}B_{1}+NS{\dot{B}}_{1},\\ u_{2}=\frac{2Rd_{2}}{\mu_{0}N}B_{2}+NS{\dot{B}}_{2}. \end{array}$$

Substituting (4) into (6), we get

(7) $$\begin{array}{c} u_{1}=\frac{R\left(3z_{1}+z_{2}\right)}{2\mu_{0}N}\cdot B_{1}+NS{\dot{B}}_{1},\\ u_{2}=\frac{R\left(z_{1}+3z_{2}\right)}{2\mu_{0}N}\cdot B_{2}+NS{\dot{B}}_{2}. \end{array}$$

From (7), we obtain

(8) $$\begin{array}{c} {\dot{B}}_{1}=\varphi_{1}=-\frac{R\left(3z_{1}+z_{2}\right)}{2\mu_{0}SN^{2}}\cdot B_{1}+\frac{1}{NS}\cdot u_{1},\\ {\dot{B}}_{2}=\varphi_{2}=-\frac{R\left(z_{1}+3z_{2}\right)}{2\mu_{0}SN^{2}}\cdot B_{2}+\frac{1}{NS}\cdot u_{2}. \end{array}$$

On all accounts, the dynamic law of maglev system can be determined by the following equations:

(9) $$\begin{array}{c} {\ddot{z}}_{1}=A\cdot \frac{B_{1}^{2}S}{\mu_{0}}+B\cdot \frac{B_{2}^{2}S}{\mu_{0}}+C\cdot f_{1}+D\cdot f_{2}+g,\\ {\ddot{z}}_{2}=B\cdot \frac{B_{1}^{2}S}{\mu_{0}}+A\cdot \frac{B_{2}^{2}S}{\mu_{0}}+D\cdot f_{1}+C\cdot f_{2}+g,\\ {\dot{B}}_{1}=\varphi_{i1}=-\frac{R\left(3z_{1}+z_{2}\right)}{2\mu_{0}SN^{2}}\cdot B_{1}+\frac{1}{NS}\cdot u_{1},\\ {\dot{B}}_{2}=\varphi_{i2}=-\frac{R\left(z_{1}+3z_{2}\right)}{2\mu_{0}SN^{2}}\cdot B_{2}+\frac{1}{NS}\cdot u_{2}. \end{array}$$

### 2.2. State-Space Equations

Choose $x={\left[x_{1},x_{2},x_{3},x_{4},x_{5},x_{6}\right]}^{T}={\left[z_{1},{\dot{z}}_{1},z_{2},{\dot{z}}_{2},B_{1},B_{2}\right]}^{T}$ as state variable vectors, and the state-space model of maglev system is given as follows:

(10) $$\begin{array}{c} {\dot{x}}_{1}=x_{2},\\ {\dot{x}}_{2}=A\cdot \frac{x_{5}^{2}S}{\mu_{0}}+B\cdot \frac{x_{6}^{2}S}{\mu_{0}}+C\cdot f_{1}+D\cdot f_{2}+g,\\ {\dot{x}}_{3}=x_{4},\\ {\dot{x}}_{4}=B\cdot \frac{x_{5}^{2}S}{\mu_{0}}+A\cdot \frac{x_{6}^{2}S}{\mu_{0}}+D\cdot f_{1}+C\cdot f_{2}+g,\\ {\dot{x}}_{5}=-\frac{R\cdot \left(3x_{1}+x_{3}\right)}{2\mu_{0}SN^{2}}x_{5}+\frac{1}{NS}u_{1},\\ {\dot{x}}_{6}=-\frac{R\cdot \left(x_{1}+3x_{3}\right)}{2\mu_{0}SN^{2}}x_{6}+\frac{1}{NS}u_{2}. \end{array}$$

Simplify (10):

(11) $$\begin{array}{c} \dot{x}=f\left(x\right)+g\left(x\right)\cdot u,\\ y=h\left(x\right), \end{array}$$

where

(12) $$\begin{array}{c} f\left(x\right)=\left[\begin{array}{c} x_{2}\\ A\cdot \frac{x_{5}^{2}S}{\mu_{0}}+B\cdot \frac{x_{6}^{2}S}{\mu_{0}}+C\cdot f_{1}+D\cdot f_{2}+g\\ x_{4}\\ B\cdot \frac{x_{5}^{2}S}{\mu_{0}}+A\cdot \frac{x_{6}^{2}S}{\mu_{0}}+D\cdot f_{1}+C\cdot f_{2}+g\\ -\frac{R\cdot \left(3x_{1}+x_{3}\right)}{2\mu_{0}SN^{2}}x_{5}\\ -\frac{R\cdot \left(x_{1}+3x_{3}\right)}{2\mu_{0}SN^{2}}x_{6} \end{array}\right],\\ g\left(x\right)=\left[g_{1}\left(x\right)\;g_{2}\left(x\right)\right]=\left[\begin{array}{cc} 0 & 0\\ 0 & 0\\ 0 & 0\\ 0 & 0\\ \frac{1}{NS} & 0\\ 0 & \frac{1}{NS} \end{array}\right],\\ h\left(x\right)=\left[\begin{array}{c} h_{1}\left(x\right)\\ h_{2}\left(x\right) \end{array}\right]=\left[\begin{array}{c} x_{1}\\ x_{3} \end{array}\right],\;\;u=\left[\begin{array}{c} u_{1}\\ u_{2} \end{array}\right]. \end{array}$$

**Theorem 1**

MIMO affine nonlinear system is described as

(13) $$\begin{array}{c} \dot{x}=f\left(x\right)+\sum_{i=1}^{m}g_{i}\left(x\right)u_{i},\\ y=h\left(x\right). \end{array}$$

Set the equilibrium point *x*_0_ ∈ *X*. The problem of MIMO nonlinear system feedback linearization will have a solution, if and only if a neighborhood *U* near *x*_0_ and *m* functions *h*_1_(*x*),…, *h*_*m*_(*x*) exists. The solution makes relative degree (*r*_1_,…, *r*_*m*_) of nonlinear system (11) satisfy *r*_1_ + *r*_2_ + ⋯+*r*_*m*_ = *n*, where *n* denotes the dimension of system and *m* stands for input dimension.

### 2.3. Demonstration of Definition 1

Define the equilibrium point of maglev system $x_{0}={\left[z_{10},{\dot{z}}_{10},z_{20},{\dot{z}}_{20},i_{10},i_{20}\right]}^{T}$, and suppose state variables *x*_1_, *x*_2_, *x*_5_, *x*_6_ ∈ *R*_+_, according to real maglev system construction. Compute the vector field as follows, by *f*(*x*) and *g*(*x*):

(14) $$\begin{array}{c} L_{g_{1}}L_{f}^{0}h_{1}\left(x\right)=0,\;\;L_{g_{2}}L_{f}^{0}h_{1}\left(x\right)=0,\\ L_{g_{1}}L_{f}h_{1}\left(x\right)=0,\;\;L_{g_{2}}L_{f}h_{1}\left(x\right)=0,\\ L_{g_{1}}L_{f}^{2}h_{1}\left(x\right)=\frac{2A\cdot x_{5}}{\mu_{0}N}\neq 0,\;\;L_{g_{2}}L_{f}^{2}h_{1}\left(x\right)=\frac{2B\cdot x_{6}}{\mu_{0}N}\neq 0,\\ L_{g_{1}}L_{f}^{0}h_{2}\left(x\right)=0,\;\;L_{g_{2}}L_{f}^{0}h_{2}\left(x\right)=0,\\ L_{g_{1}}L_{f}h_{2}\left(x\right)=0,\;\;L_{g_{2}}L_{f}h_{2}\left(x\right)=0,\\ L_{g_{1}}L_{f}^{2}h_{2}\left(x\right)=\frac{2B\cdot x_{5}}{\mu_{0}N}\neq 0,\;\;L_{g_{2}}L_{f}^{2}h_{2}\left(x\right)=\frac{2A\cdot x_{6}}{\mu_{0}N}\neq 0. \end{array}$$

Namely, above equations satisfy

(15) LgjLfkhi(x)=0,   ∀x∈V,  1≤j≤m,  1≤i≤m,0≤k≤ri−2,LgjLfrihi(x)≠0,

where

(16) H(x)=[Lg1Lfr1−1h1(x)Lg2Lfr1−1h1(x)Lg1Lfr2−1h2(x)Lg2Lfr2−1h2(x)]=[2A·x5μ0N2B·x6μ0N2B·x5μ0N2A·x6μ0N].

At the moment, det⁡  *H*(*x*) = 4(*A* ^2^ − *B* ^2^) · *x*_5_ *x*_6_/*μ*_0_^2^ *N* ^2^ ≠ 0; then *E*(*x*) is not singular. Relative degree of maglev system is (*r*_1_, *r*_2_) = (3,3), and

(17) $$\begin{array}{c} r=\sum_{i=1}^{2}r_{i}=6=n. \end{array}$$

Therefore, Theorem 1 has been proved.

### 2.4. MIMO Double Suspension Points Model with Feedback Linearization

Compute the vector field generated by *f*(*x*) and *h*(*x*) as follows:

(18) $$\begin{array}{c} L_{f}^{3}h_{1}\left(x\right)=\frac{1}{\mu_{0}^{2}N^{2}}\left[-AR\left(3x_{1}+x_{3}\right)x_{5}^{2}-BR\left(x_{1}+3x_{3}\right)x_{6}^{2}\right],\\ L_{f}^{3}h_{2}\left(x\right)=\frac{1}{\mu_{0}^{2}N^{2}}\left[-BR\left(3x_{1}+x_{3}\right)x_{5}^{2}-AR\left(x_{1}+3x_{3}\right)x_{6}^{2}\right]. \end{array}$$

Design feedback control value *u* = *H* ^−1^(*x*)[−*b*(*x*) + *v*], where the terms *u* = [*u*_1_ *u*_2_]^*T*^, *v* = [*v*_1_ *v*_2_]^*T*^, and

(19) $$\begin{array}{c} H^{-1}\left(x\right)=\frac{\mu_{0}N}{2\left(A^{2}-B^{2}\right)}\cdot \left[\begin{array}{cc} \frac{A}{x_{5}} & -\frac{B}{x_{6}}\\ -\frac{B}{x_{5}} & \frac{A}{x_{6}} \end{array}\right],\\ b\left(x\right)={\left[L_{f}^{r_{1}}h_{1}\left(x\right)\;L_{f}^{r_{2}}h_{2}\left(x\right)\right]}^{T}={\left[L_{f}^{3}h_{1}\left(x\right)\;L_{f}^{3}h_{2}\left(x\right)\right]}^{T}. \end{array}$$

We can obtain the control value after linearization from (19):

(20) u=H−1(x)[−b(x)+v]={μ0N2(A2−B2)·[−Ax5·Lf3h1(x)+Bx6·Lf3h2(x)+Ax5·v1−Bx6·v2]μ0N2(A2−B2)·[Bx5·Lf3h1(x)−Ax6·Lf3h2(x)−Bx5·v1+Ax6·v2].

The diffeomorphic mapping matrix is

(21) Φ(x)=[h1(x)Lfh1(x)Lf2h1(x)h2(x)Lfh2(x)Lf2h2(x)]=[x1x2A·x52Sμ0+B·x62Sμ0+C·f1+D·f2+gx3x4B·x52Sμ0+A·x62Sμ0+D·f1+C·f2+g].

Choose the coordinates of transformation by matrix Φ(*x*):

(22) δ=[h1(x)Lfh1(x)Lf2h1(x)h2(x)Lfh2(x)Lf2h2(x)]T.

In sum, maglev control system model after linearization is showed as follows:

(23) $$\begin{array}{c} \dot{\delta }=\Psi \delta +Tv,\\ y=\Omega \delta , \end{array}$$

where

(24) $$\begin{array}{c} \Psi =\left[\begin{array}{cccccc} 0 & 1 & 0 & 0 & 0 & 0\\ 0 & 0 & 1 & 0 & 0 & 0\\ 0 & 0 & 0 & 0 & 0 & 0\\ 0 & 0 & 0 & 0 & 1 & 0\\ 0 & 0 & 0 & 0 & 0 & 1\\ 0 & 0 & 0 & 0 & 0 & 0 \end{array}\right],\;\;T=\left[\begin{array}{cc} 0 & 0\\ 0 & 0\\ 1 & 0\\ 0 & 0\\ 0 & 0\\ 0 & 1 \end{array}\right],\\ \Omega =\left[\begin{array}{cccccc} 1 & 0 & 0 & 0 & 0 & 0\\ 0 & 0 & 0 & 1 & 0 & 0 \end{array}\right]. \end{array}$$

Maglev system after feedback linearization is expressed by two-level integral subsystems:

(25) $$\begin{array}{c} G\left(s\right)=\left[\begin{array}{cc} \frac{1}{s^{3}} & 0\\ 0 & \frac{1}{s^{3}} \end{array}\right]. \end{array}$$

**Figure 3:**
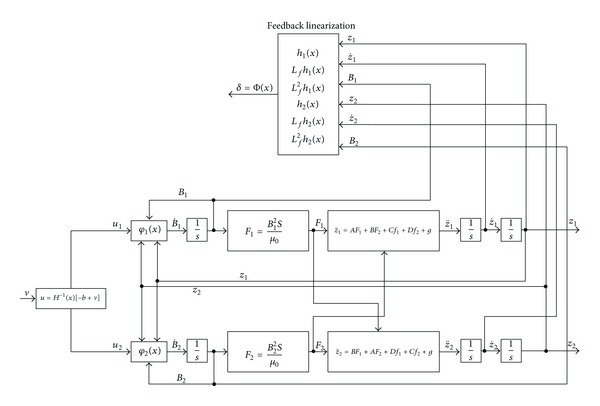
Double suspension points' control construction diagram with feedback linearization.

Double suspension points' closed control block diagram with magnetic flux feedback is showed in Figure 3.

## 3. Design of Suspension Controller

Controlled matrix of system (23) is

(26) Mc=[TΨTΨ2TΨ3TΨ4TΨ5T]=[000010000000001000000000100000000000000001000000000100000000010000000000].

Because of rank⁡  (*M*_*c*_) = 6, maglev system after linearization is controlled completely. So we design double suspension controller for regulating control properties of suspension system:

(27) $$\begin{array}{c} v_{1}=K_{p1}\cdot \left(d_{1}-z_{0}\right)+K_{d1}\cdot {\dot{d}}_{1}+K_{i1}\cdot \int \left(d_{1}-z_{10}\right)\cdot dt,\\ v_{2}=K_{p2}\cdot \left(d_{2}-z_{0}\right)+K_{d2}\cdot {\dot{d}}_{2}+K_{i2}\cdot \int \left(d_{2}-z_{20}\right)\cdot dt. \end{array}$$

The geometrical relationship between measured positions and real physical positions in (28) is as follows:

(28) $$\begin{array}{c} d_{1}=\frac{3}{4}z_{1}+\frac{1}{4}z_{2},\\ d_{2}=\frac{1}{4}z_{1}+\frac{3}{4}z_{2}. \end{array}$$

We can get

(29) v1=Kp1·(34z1+14z2−z10)+Kd1·z˙1 +Ki1·∫(34z1+14z2−z10)·dt,v2=Kp2·(14z1+34z2−z0)+Kd2·z˙2 +Ki2·∫(14z1+34z2−z20)·dt.

**Figure 4:**
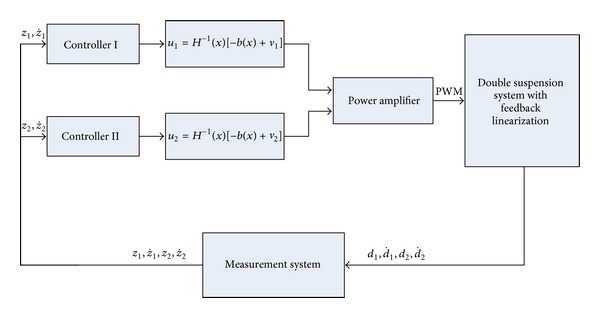
Block diagram of double suspension points system.

By now, double suspension points' controller based on magnetic flux feedback was completed. Construction of the controller is described in Figure 4.

## 4. Experiments

**Figure 5:**
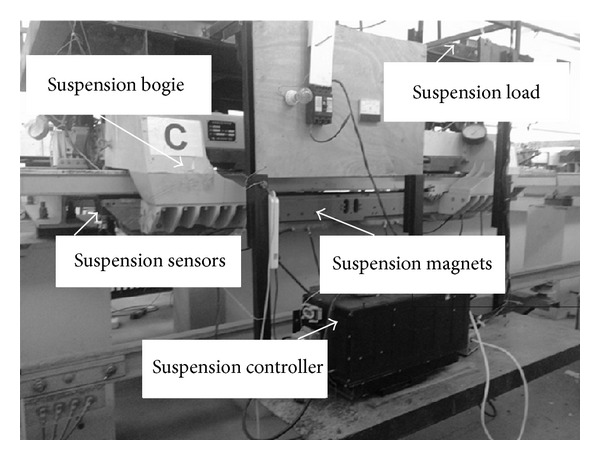
CMS04 maglev control experiment platform based on magnetic flux feedback.

Some experiments have been completed on CMS04 suspension bogie designed by NUDT (National University of Defense Technology), which is showed in Figure 5. These experiments conclude experiment S1 and experiment S2. Algorithm S1: traditional position-current double cascade suspension PID control method. Algorithm S2: magnetic flux feedback control algorithm based on MIMO feedback linearization. Initial suspension gap of maglev system is 25 mm, and the set value is 10 mm. Decoupling control experiment has been implemented on CMS04 maglev control experiment platform.

Standard maglev bogie's parameters are given in Table 2.

**Figure 6:**
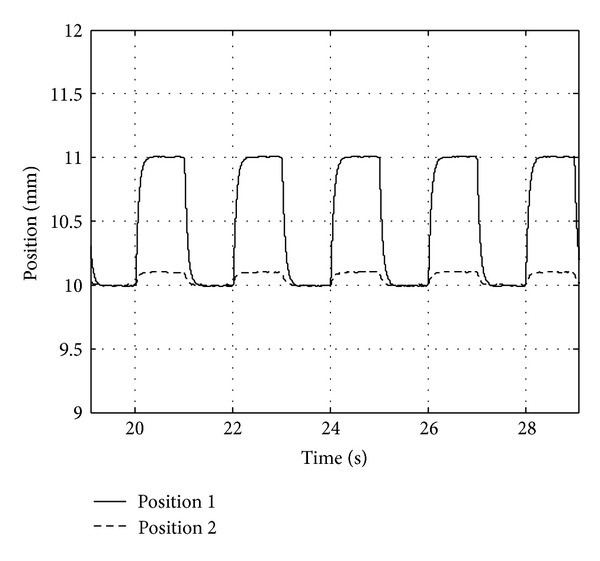
Position curves of algorithm S1.

**Figure 7:**
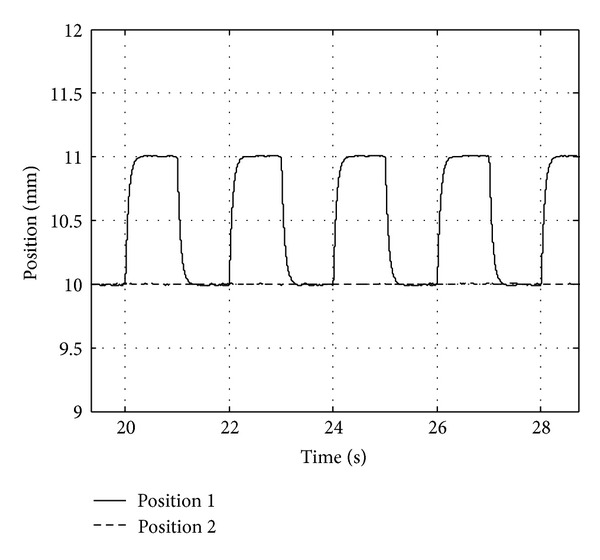
Position curves of algorithm S2.

Decoupling property test comes true on one suspension point of maglev bogie. Set expected position 10 mm. When maglev system becomes stable, we add additional square signal with amplitude 0.5 mm and period 4 s. Observe suspension signals with two control algorithms S1 and S2. Experiment results are showed in Figures 6 and 7.

Experiment results illustrate that when double suspension control system applies algorithm S2, the dynamic decoupling problem has been worked out. S2 raises the robustness and stability properties of maglev control system, but method S1 has no decoupling effect. 

## 5. Conclusions

Double suspension control model has been founded with magnetic flux signal based on MIMO feedback linearization. The feedback linearization algorithm enables accurate linearizing model to keep all properties of original nonlinear system which overcome the disadvantages of Taylor's expansion linearization method. In order to work out dynamic coupling problem and external interference problem of EMS mid-low-speed maglev vehicle, we take magnetic flux signal back to maglev control system and design double suspension compensable controller. Some experiments about new algorithm have been done in maglev vehicle CMS04 designed by NUDT. Experiment results demonstrate that double suspension module is precise based on MIMO state feedback linearization theory. With magnetic flux feedback, the maglev control system has better robustness and adaptability than traditional algorithm.

## Figures and Tables

**Table 1:** Symbols in double suspension points system.

Signs	Signification
*N*	Number of magnetic coils
*R*	Magnetic resistance
μ_0_	Magnetic permeability of atmosphere 4π × 10^−7^ H/m
*g*	Acceleration of gravitation 9.8 m/s^2^
*θ*	Separation angle between electromagnet axis line and horizon
*F*_1_	Electromagnetic force of suspension point *P*_1_
*N*_1_	Disturbing force of suspension point *P*_1_
*L*	Total length of electromagnet
*i*_1_(*t*)	Current of magnetic coil in suspension *P*_1_
*u*_1_(*t*)	Voltage of magnetic coil in suspension point *P*_1_
*z*_1_(*t*)	Measured position value in suspension point *P*_1_
*d*_1_(*t*)	Average gap value between guideway and suspension point *P*_1_
*S*	Magnetic pole area
*B*	Suspension gap flux density
*M*	Total mass of suspension system
*I*	Rotation inertia of electromagnet for *O* point
*d*	Distance from guideway to *O* point
*F*_2_	Electromagnetic force of suspension point *P*_2_
*N*_2_	Disturbing force of suspension point *P*_2_
*l*	Disturbing force of arm relative to *O* point
*i*_2_(*t*)	Current of magnetic coil in suspension point *P*_2_
*u*_2_(*t*)	Voltage of magnetic coil in suspension point *P*_2_
*z*_2_(*t*)	Measured position value in suspension point *P*_2_
*d*_2_(*t*)	Average gap value between guideway and suspension point *P*_2_

**Table 2:** Standard maglev bogie parameters of CMS04 maglev vehicle.

Parameters	Values
*m* [kg]	653
*N* [integer]	324
*A* [m^2^]	0.0235
*R* [Ω]	0.5
*z*_0_/*m*	0.008
*B*_0_/*T*	23
*g*	9.8
*μ*_0_	4*π* × 10^−7^
